# Interferon-armed RBD dimer enhances the immunogenicity of RBD for sterilizing immunity against SARS-CoV-2

**DOI:** 10.1038/s41422-021-00531-8

**Published:** 2021-07-15

**Authors:** Shiyu Sun, Yueqi Cai, Tian-Zhang Song, Yang Pu, Lin Cheng, Hairong Xu, Jing Sun, Chaoyang Meng, Yifan Lin, Haibin Huang, Fang Zhao, Silin Zhang, Yu Gao, Jian-Bao Han, Xiao-Li Feng, Dan-Dan Yu, Yalan Zhu, Pu Gao, Haidong Tang, Jincun Zhao, Zheng Zhang, Jiaming Yang, Zhenxiang Hu, Yang-Xin Fu, Yong-Tang Zheng, Hua Peng

**Affiliations:** 1 Key Laboratory of Infection and Immunity, Institute of Biophysics, Chinese Academy of Sciences, Beijing, China; 2 University of Chinese Academy of Sciences, Beijing, China; 3 Key Laboratory of Animal Models and Human Disease Mechanisms of the Chinese Academy of Sciences, Kunming Institute of Zoology, Chinese Academy of Sciences, Kunming, Yunnan China; 4 Institute of Basic Medical Sciences, Chinese Academy of Medical Sciences, Beijing, China; 5 Institute for Hepatology, National Clinical Research Center for Infectious Disease, Shenzhen Third People’s Hospital, Shenzhen, Guangdong Province China; 6 State Key Laboratory of Respiratory Disease, Guangzhou Institute of Respiratory Health, the First Affiliated Hospital of Guangzhou Medical University, Guangzhou, Guangdong China; 7 LivzonBio, Inc., Zhuhai, Guangdong China; 8 School of Pharmaceutical Sciences, Tsinghua University, Beijing, China; 9 Key Laboratory of Protein and Peptide Pharmaceuticals, Institute of Biophysics, Chinese Academy of Sciences, Beijing, China; 10 Kunming National High-level Biosafety Research Center for Non-human Primates, Center for Biosafety Mega-Science, Kunming Institute of Zoology, Chinese Academy of Sciences, Kunming, Yunnan China; 11 Department of Pathology, University of Texas Southwestern Medical Center, Dallas, TX USA; 12 Bioland Laboratory (Guangzhou Regenerative Medicine and Health Guangdong Laboratory), and Guangzhou Laboratory, Guangzhou, China

**Keywords:** Innate immunity, Biological techniques

## Abstract

Severe acute respiratory syndrome coronavirus 2 (SARS-CoV-2) has caused a global crisis, urgently necessitating the development of safe, efficacious, convenient-to-store, and low-cost vaccine options. A major challenge is that the receptor-binding domain (RBD)-only vaccine fails to trigger long-lasting protective immunity if used alone for vaccination. To enhance antigen processing and cross-presentation in draining lymph nodes (DLNs), we developed an interferon (IFN)-armed RBD dimerized by an immunoglobulin fragment (I-R-F). I-R-F efficiently directs immunity against RBD to DLNs. A low dose of I-R-F induces not only high titers of long-lasting neutralizing antibodies (NAbs) but also more comprehensive T cell responses than RBD. Notably, I-R-F provides comprehensive protection in the form of a one-dose vaccine without an adjuvant. Our study shows that the pan-epitope modified human I-R-F (I-P-R-F) vaccine provides rapid and complete protection throughout the upper and lower respiratory tracts against a high-dose SARS-CoV-2 challenge in rhesus macaques. Based on these promising results, we have initiated a randomized, placebo-controlled, phase I/II trial of the human I-P-R-F vaccine (V-01) in 180 healthy adults, and the vaccine appears safe and elicits strong antiviral immune responses. Due to its potency and safety, this engineered vaccine may become a next-generation vaccine candidate in the global effort to overcome COVID-19.

## Introduction

The COVID-19 pandemic, caused by severe acute respiratory syndrome coronavirus 2 (SARS-CoV-2), has swept across the world since the outbreak in late 2019.^1^ Mutant coronaviruses continue to evolve, some with improved receptor-binding affinity and infectivity. Although various categories of vaccine candidates against SARS-CoV-2 have been developed, improved vaccines are still urgently needed for public health and various socioeconomic crises. In addition to having long-lasting potency, vaccines should be stable in 4–25 °C storage, easy to produce, inexpensive, and safe for all ages to be available for the most affected, densely populated, and underresourced countries worldwide.

The leading vaccine candidates are mRNA-based, inactivated, or adenovirus (AdV)-based vaccines (https://vac-lshtm.shinyapps.io/ncov_vaccine_landscape/). Inactivated vaccines can be made by traditional methods. Antibodies induced by inactivated vaccines target all viral proteins, which are mostly unrelated to neutralization. Natural monomeric spike (S) protein or receptor-binding domain (RBD) yields low titers of neutralizing antibodies (NAbs) due to their poor immunogenicity.^2–4^ Some studies have described modified S or RBD, such as S trimer and RBD dimer, which were developed to generate more NAbs than monomeric proteins.^5,6^ Additionally, RBD fused with the Fc domain showed potentially greater immune effects than RBD.^7–9^ Alum adjuvant is used in most of these viral antigen vaccines to induce stronger humoral immunity preferentially, but does not promote T cell responses, especially type 1 T helper (Th1) cell and cytotoxic T lymphocyte (CTL) responses.^2–4^ Although novel adjuvants may be more potent, they are challenging to prepare and increase the risk of severe side effects, resulting in limited usage.^5,10,11^ Recombinant vaccines based on AdV vectors, such as Ad5-nCoV, stimulated both B cell and T cell responses. However, ubiquitous pre-existing anti-vector immunity may disrupt immune responses, resulting in low NAb titers in trials and an ineffective immune boost after repeated vaccination.^12–14^ mRNA-based vaccines are currently the leading vaccines due to their ability to be rapidly manufactured after new outbreaks and to induce moderate to strong antibody responses and T cell responses. However, it remains unclear whether the reactogenicity of certain mRNA vaccines varies based on age and race. The strict conditions for the preservation and transportation of mRNA vaccines further limit their broader application, especially in developing countries.^15–18^

To increase the immunogenicity of RBD, we developed a next-generation fusion protein vaccine named I-R-F in which RBD is armed with interferon-α (IFNα) at the N terminus and is dimerized by human IgG1 Fc at the C terminus. This allows the targeting and activation of dendritic cells (DCs) in lymph nodes (LNs). Armed with IFN and dimerized by Fc, which enhances antigen processing and presentation, low-dose I-R-F showed more potent immunogenicity than monomeric RBD and induced robust antibody titers with balanced IgG1 and IgG2a subtypes and robust CD8^+^ T cell responses, even without an additional adjuvant. We further added a pan HLA-DR-binding epitope (PADRE) to I-R-F (named I-P-R-F) to enhance helper T cell responses.^19^ I-P-R-F, intramuscularly (i.m.) injected in the lateral thigh, effectively provided complete protection in both the upper and lower respiratory tracts against a high-titer SARS-CoV-2 challenge in rhesus macaques. Therefore, IFN-armed RBD dimer fusion proteins may be potent COVID-19 vaccine candidates. This strategy could also be expanded to various infectious diseases, serving as a promising technology platform for vaccine development.

## Results

### I-R-F initiates highly efficient antigen presentation and creates a favorable environment for antibody generation

**Fig. 1:**
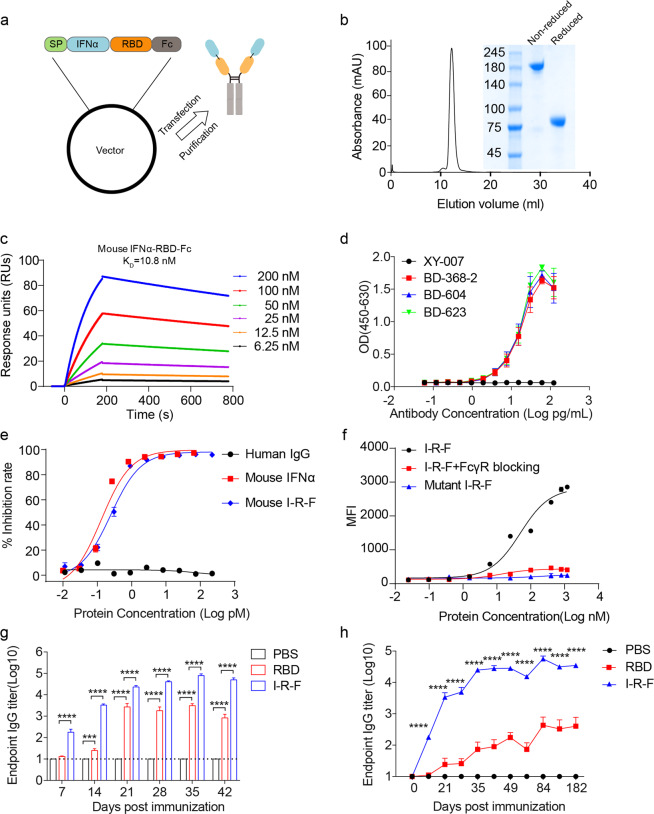
IFN-armed I-R-F induces robust IgG response. **a** Schematic diagram of the I-R-F fusion protein. The structural elements contain a mouse IFNα4a (IFNα), receptor-binding domain (RBD), immunoglobulin Fc fragment (Fc). **b** Size exclusion chromatography of I-R-F was performed on a Superdex200 Increase Column. The ultraviolet absorption at 280 mm is shown. The insert photograph presents the SDS-PAGE of the eluted protein samples. **c** The real-time binding kinetics between I-R-F and hACE2 was determined by the BIAcore T100 system. **d** Evaluation of the binding ability of monoclonal antibodies to I-R-F by ELISA. To determine the known RBD epitopes in I-R-F, ELISA plate was coated with I-R-F. Then, 2-fold serially diluted monoclonal antibodies were detected. The anti-Pres1 XY007 monoclonal antibody was used as control. The absorbance was read at 450–630. **e** The bioactivity of IFNα contained in I-R-F was measured by an anti-viral infection biological assay. **f** Binding of mouse I-R-F to FcγR on RAW264.7 cells. Cells were incubated with serial dilutions of I-R-F, I-R-F mixed with anti-FcγR, or I-R-F with mutant Fc, followed by incubation with fluorophore-conjugated anti-human IgG secondary antibody. The mean fluorescence intensity (MFI) was measured by flow cytometry (*n* = 3). **g** BALB/c mice (*n* = 8/group) were immunized i.m. with 10 μg of I-R-F or equimolar RBD protein, mixed with alum adjuvant, respectively. Mice were re-immunized with the same dose of vaccine on day 14 post the first shot. PBS containing alum adjuvant was used as a negative control. Sera were collected on days 7, 14, 21, 28, 35, and 42 after initial immunization, and the IgG levels were measured by ELISA. **h** Groups of BALB/c mice (*n* = 7/groups) were i.m. immunized twice on day 0 and day 14 with 10 μg of alum-adjuvanted I-R-F, or equimolar RBD or alum alone as a control. Serum was collected at indicated time points, and the kinetics of the RBD-specific IgG antibody titers were determined by ELISA. The dashed line indicates the limit of detection. The data shown are presented as mean ± SEM. *P* values were determined by one-way ANOVA with multiple comparison tests. ***P < 0.001, ****P < 0.0001.

The RBD of SARS-CoV-2 is the primary viral protein domain that initiates cell entry and is the major target of NAbs. Similar to a previous study,^5^ we found that RBD was weakly immunogenic and induced very low titers of anti-RBD antibodies, even with alum adjuvant. RBD could be poorly immunogenic because its molecular size is small for antigen presentation by antigen-presenting cells (APCs), it lacks epitopes for T cell responses, and it is unstable in vivo. Organized lymphoid nodes are sites that are essential for improving antigen presentation and interaction among DCs and various lymphoid cells.^20,21^ RBD may be too small to effectively enter lymph vessels before diffusing to the surrounding muscular tissues. Therefore, we constructed an RBD-Fc fusion protein, in which RBD is dimerized via Fc of human Ig, which can increase protein stability and size to promote effective LN targeting and FcR^+^ DC capture. To further increase antigen processing and presentation by DCs, we armed the RBD with type I IFN by fusing mouse IFNα at the N terminus of RBD-Fc to form a natural dimer, named I-R-F (Fig. 1a). I-R-F was expressed at high levels in 293 F cells and easily purified from the supernatant with a protein-A Sepharose column, as previously described.^22^ After a one-step protein-A column purification, a single peak of intact I-R-F fusion protein was obtained using size exclusion chromatography. Additionally, SDS-PAGE confirmed that the purified fusion protein was the correct size (Fig. 1b). Real-time binding kinetics showed that I-R-F had a high binding affinity for hACE2 (K_D_ = 10.8 nM, as determined by a BIAcore T100 system), suggesting that RBD in the I-R-F fusion protein was efficiently exposed (Fig. 1c). To further evaluate the antigen epitope exposure of RBD in the I-R-F fusion protein, we used three RBD-specific monoclonal antibodies (MAbs) (BD-368-2, BD-604, and BD-623) to detect the epitopes corresponding to the antibodies. BD-368-2, BD-604 and BD-623 were all isolated from convalescent SARS-CoV-2 patients and specifically recognized the SARS-CoV-2 RBD. BD-368-2 recognizes the epitope in the far corner of RBD regardless of the spatial conformation.^23,24^ BD-604 belongs to the class 1 NAbs, which bind to the conformational epitopes overlapping with the RBD–ACE2 binding interface and bind only to up RBDs, not down RBDs.^23,25^ BD-623 is a class 2 NAb with a long CDRH3, which recognizes that the epitopes that overlap with the ACE2 binding site and can bind to up and down RBDs.^25,26^ The results showed that all three MAbs bound to I-R-F with high affinity, indicating that there was sufficient exposure of RBD epitopes on the I-R-F vaccine molecule (Fig. 1d). The IFN bioactivity of I-R-F was examined by an antiviral infection assay. I-R-F inhibited VSV-GFP infection in L929 cells in a dose-dependent manner (Fig. 1e). The function of the Fc domain was determined by RAW264.7 cell binding assays, which showed the binding capacity of I-R-F to APCs (Fig. 1f).

To compare the immunogenicity of RBD and I-R-F, mice were vaccinated with 10 μg I-R-F or equimolar RBD protein formulated with alum adjuvant using a prime-boost vaccination schedule on days 0 and 14, respectively. We observed a much higher RBD-specific IgG response in the I-R-F-vaccinated group than in the RBD-vaccinated group. Immune responses were induced quickly in the I-R-F groups, and viral-specific IgG was detected as early as 7 days after immunization. In contrast, the sera collected from the RBD groups presented a much weaker and more delayed antibody response (Fig. 1g). The long-lasting and strong antibody response has been maintained for more than 6 months (Fig. 1h). Moreover, I-R-F induced stronger humoral immunity than equimolar RBD dimer and RBD-Fc (Supplementary information, Fig. S1a). IFNα has been widely used in clinical treatment, which is also used as an adjuvant for vaccines.^27^ To determine whether the use of IFNα as an adjuvant instead of I-R-F could also induce a robust immune response, a mixture of RBD-Fc plus IFNα was compared with I-R-F. As shown in Supplementary information, Fig. S1b, enhanced humoral immunity was induced in the mixture group than in the R-F only group, but it was not equal to that in the I-R-F group. Then, mice were immunized twice either i.m. or subcutaneously with 10 μg of alum-adjuvanted I-R-F. We found that both i.m. and subcutaneous immunizations generated strong and long-lasting IgG responses (Supplementary information, Fig. S1c).

The potential adverse effects of I-R-F vaccination were then examined. There were no obvious body weight changes observed, even in mice immunized with an extremely high dose (100 μg) I-R-F (Supplementary information, Fig. S2a). Moreover, only IL-6 slightly increased among inflammatory cytokines (IL-12, IFNγ, IL-6, TNF-α, and IL-10) in mice immunized with 50 or 100 μg I-R-F (Supplementary information, Fig. S2b). Neither ALT nor AST was elevated in any group (Supplementary information, Fig. S2c). Furthermore, no IFNα-specific antibodies were detected in mice immunized with I-R-F (Supplementary information, Fig. S2d). Therefore, I-R-F overcomes the poor immunogenicity of monomeric RBD, further improves the immunogenicity of RBD dimer and RBD-Fc, and induces long-lasting NAbs without side effects.

### A single or low dose of I-R-F induces robust NAbs even with no additional adjuvant

**Fig. 2:**
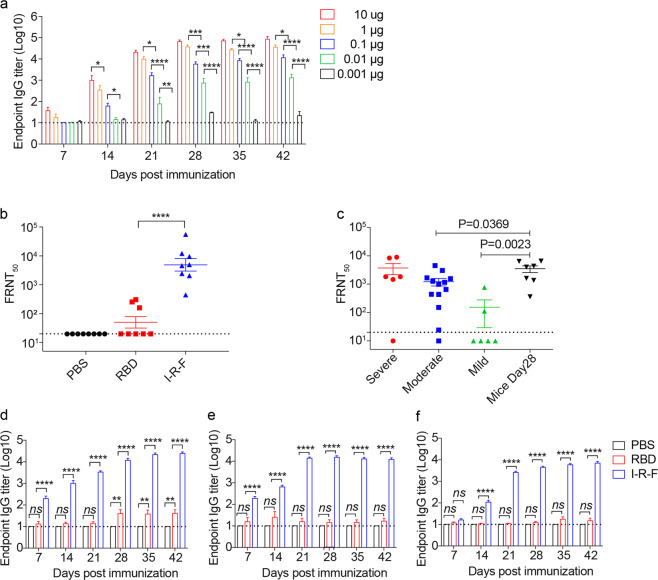
I-R-F induces robust neutralization antibodies with low-dose, single-dose, and without adjuvant. **a** The kinetics of the RBD-specific IgG antibody response. BALB/c mice (*n* = 7/group) were immunized with a 1:10 serial dilutions of the vaccine, containing 10 μg, 1 μg, 0.1 μg, 0.01 μg, 0.001 μg of I-R-F, respectively. Sera were collected to assess the levels of RBD-specific IgG. **b** The serum described in Fig. 1e on day 28 was used to determine the neutralization activity with live SARS-CoV-2 by FRNT. The sera from different groups were serially diluted and mixed with 600 FFU of SARS-CoV-2, and the mixtures were then transferred to Vero E6 cells. The number of SARS-CoV-2 foci was counted the next day. The FRNT_50_ was defined as the reciprocal of serum dilution, which inhibits 50% of viral infection. **c** Comparison of the neutralizing antibody titers in sera between the I-R-F-vaccinated mice (*n* = 7) and the convalescent COVID-19 patients with different severity (*n* = 6–13/group). The sera from 3 groups of COVID-19 convalescent patients and the I-P-F immunized mice as mentioned above in Fig. 1f were serially diluted and mixed with live SARS-CoV-2. The mixtures were added to Vero E6 cells. The neutralizing titers were presented by FRNT_50_, which was the reciprocal of serum dilution neutralizing 50% of viral infection. **d** Mouse RBD-specific IgG induced by single immunization. Mice (*n* = 8/group) were i.m. immunized once with alum-adjuvanted 10 ug I-R-F (*n* = 8), equimolar RBD protein, or alum alone, respectively. The levels of RBD-specific IgG in sera on days 7, 14, 21, 28, 35, and 42 after the first immunization were determined by ELISA. **e** Mouse RBD-specific IgG induced by vaccines without adjuvant. Mice (*n* = 7 or 8) were vaccinated with no adjuvant 10 μg I-R-F, equimolar RBD protein, or PBS control and boosted with the same dose 14 days after the initial immunization. Sera were collected every week after immunization and used to determine the IgG titers. **f** BALB/C mice (*n* = 8/group) were immunized i.m. with alum-adjuvanted 0.1 μg I-R-F, equimolar RBD protein, or alum alone, respectively, and boosted with the same dose at a 14-day interval. Sera were collected on days 7, 14, 21, 28, 35, and 42 after the initial immunization. RBD-specific IgG levels were analyzed by ELISA. The dashed line indicates the limit of detection. Data are shown as mean ± SEM. *P* values were calculated by one-way ANOVA with multiple comparison tests. ns, not significant, *P < 0.05, **P < 0.01, ***P < 0.001, ****P < 0.0001.

To determine the appropriate doses for long-lasting antibody production, mice were i.m. immunized with varying doses of alum-adjuvanted I-R-F (from 10 μg to 0.001 μg) on days 0 and 14. Viral-specific IgG was induced by I-R-F in a dose-dependent manner. Impressively, I-R-F, even at the low dose of 0.01 μg, generated strong and long-lasting IgG responses (Fig. 2a). Sera from immunized mice were collected to determine the NAb titers with live SARS-CoV-2 by a focus-reduction neutralization test (FRNT). The I-R-F group presented a much higher titer of NAbs than the RBD group. Even if mice were immunized twice with a high dose of RBD (10 μg), they only generated moderate levels of antibodies to RBD (Fig. 1g), which failed to block virus infection in vitro (Fig. 2b). These results indicate that some of the IgG detected in RBD-vaccinated mice was non-NAb, and a certain level of antibody binding to RBD does not necessarily lead to viral neutralization. I-R-F-vaccinated mice generated higher NAb titers than most convalescent COVID-19 patients, especially patients with mild to moderate symptoms. Some patients with mild symptoms had no detectable NAbs (Fig. 2c). To further evaluate I-R-F’s potency, mice were i.m. immunized with only one dose of alum-adjuvanted I-R-F (10 μg) or an equimolar dose of RBD. Strong and durable anti-RBD-specific IgG was detected only in I-R-F-vaccinated mice but not in RBD-vaccinated mice (Fig. 2d). We further compared traditional double- or triple-dose immunization with one-dose administration in Supplementary information, Fig. S1d. A single dose of I-R-F induced strong and long-lasting IgG responses, similar to repeated immunization (Supplementary information, Fig. S1d).

To determine whether IFNα could enhance the immunogenicity of RBD in the absence of adjuvant, mice were i.m. immunized with 10 μg of I-R-F or equimolar RBD protein without alum. Again, robust and long-lasting RBD-specific IgG was detected merely in the I-R-F without adjuvant group but not in the RBD group (Fig. 2e), indicating that IFNα in the fusion protein functions as a natural adjuvant to enhance vaccine-induced immune responses. Furthermore, mice were i.m. immunized twice (day 0 and 14) with low-dose (0.1 μg) alum-adjuvanted I-R-F or equimolar RBD. The RBD group failed to produce detectable RBD-specific IgG, while the I-R-F group produced high levels of long-lasting antibodies to RBD (Fig. 2f). Impressively, we observed that a single dose of I-R-F, even when used without adjuvant or at a low dose, also generated high titers of NAbs, as shown in the pseudovirus neutralization assay (Supplementary information, Fig. S1e). These results demonstrate that I-R-F could be a potent antiviral vaccine even under unfavorable conditions, such as with the administration of single injection, no adjuvant, or trace amounts of antigen.

### I-R-F induces SARS-CoV-2-specific B cell, Th1, Th2, and CD8^+^ T cell immune responses

**Fig. 3:**
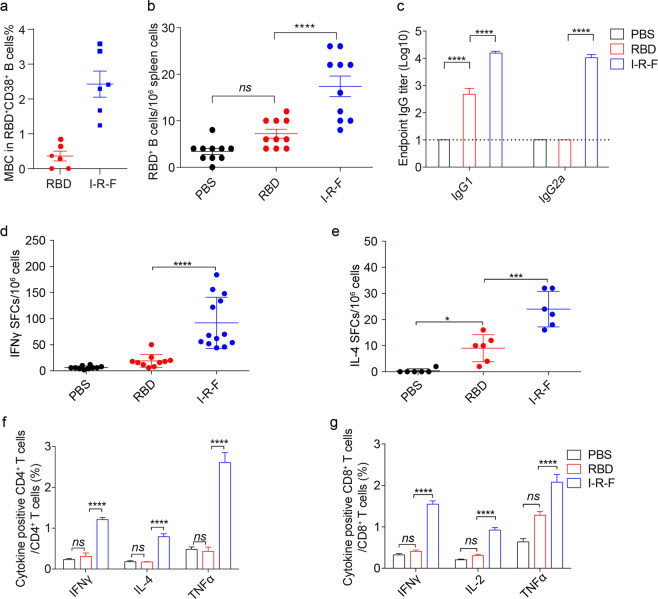
I-R-F induces SARS-CoV-2 specific Th1, Th2, and CD8^+^ T cell immune responses. **a**, **b** C57BL/6 mice were immunized with 10 μg I-R-F, equimolar RBD, or PBS with a prime-boost vaccination regimen in a 14-day interval. Mice were sacrificed six months post the first vaccination, and splenocytes were collected. **a** ELISPOT assay was performed to determine RBD-specific B cells in the spleen. **b** The percentage of memory B cells in the spleen was analyzed. **c** BALB/c mice (*n* = 8/group) were immunized with 10 μg of I-R-F, equimolar RBD, or PBS twice in a 14-day interval. PBS was used as a negative control. Sera were collected on day 28 after the initial immunization and used to determine the IgG subclasses. **d**–**g** C57BL/6 mice were immunized with 10 μg I-R-F, equimolar RBD, or PBS with a prime-boost vaccination regimen in a 14-day interval. Mice were sacrificed, and splenocytes were collected 28 days post the first vaccination. ELISPOT assay was performed for IFNγ (**d**) and IL-4 (**e**) secretion from mice splenocytes stimulated with RBD peptide pool. Splenocytes were incubated with an RBD peptide pool. **f** The percentages of IFNγ^+^, IL-4^+^, and TNFα^+^ CD4^+^T cells were determined by ICCS. **g** The percentages of IFNγ^+^, IL-2^+^, and TNFα^+^ CD8^+^T cells were determined by ICCS. The dashed line indicates the limit of detection. Data are presented as mean ± SEM. *P* values in (**a**–**e**) were calculated by one-way ANOVA with multiple comparison tests. *P* values in (**f**–**g**) were calculated by two-way ANOVA with multiple comparison tests. ns, not significant, ***P < 0.001, ****P < 0.0001.

Protein antigens with alum adjuvant often preferentially generate Th2-biased antibody responses instead of strong Th1 and CD8^+^ T cell responses.^28,29^ To examine antigen-specific memory B cells, we performed a B cell enzyme-linked immunospot (ELISPOT) assay three months after immunization and observed a significantly higher number of RBD-specific B cells in the I-R-F group than the RBD group (Fig. 3a, b; Supplementary information, Fig. S3a, b). IgG1 is associated with Th2 responses in mice, and IgG2a often indicates Th1 responses.^30,31^ To determine which type of immune responses was induced by I-R-F, mice were immunized with 10 μg of I-R-F or equimolar RBD in alum, and the different subtypes of IgG were determined by Enzyme-Linked Immunosorbent Assay **(**ELISA). I-R-F induced not only IgG1 (Th2) responses but also IgG2a (Th1) responses, while RBD only generated IgG1 responses (Fig. 3c). To further confirm the subtype of T cell responses, splenocytes from immunized mice were collected and stimulated with a peptide pool of SARS-CoV-2 RBD. Both CD4^+^ and CD8^+^ T cells and IFN-γ- and IL-4-secreting cells were detected. The I-R-F groups produced strong Th1 and Th2 responses, while the RBD groups exhibited only weak IL-4 responses and no IFN-γ responses (Fig. 3d, e; Supplementary information, Fig. S3c, d).

The proportion of CD4^+^ T cells that produced IFN-γ, IL-4, and TNFα was determined by intracellular cytokine staining. CD4^+^ T cells in the I-R-F-vaccinated groups produced all three cytokines, while CD4^+^ T cells in the RBD-vaccinated groups produced none of these cytokines, similar to the unimmunized group (Fig. 3f; Supplementary information, Fig. S3e). To determine whether CD8^+^ T cells respond to viral-specific RBD, intracellular cytokine staining was performed to determine the proportion of IFN-γ-producing CD8^+^ T cells after stimulation with the RBD peptide pool. Notably, I-R-F induced a much higher number of cytokine-producing CD8+ T cells in vaccinated mice (Fig. 3g; Supplementary information, Fig. S3f), suggesting that the poor immunogenicity of RBD may not result from a lack of proper T and B cell epitopes. Therefore, I-R-F can induce potent and comprehensive T and B cell responses, addressing the main challenge of protein-based COVID-19 vaccines.

### I-R-F efficiently stimulates Tfh and GC generation by targeting DCs in LNs

**Fig. 4:**
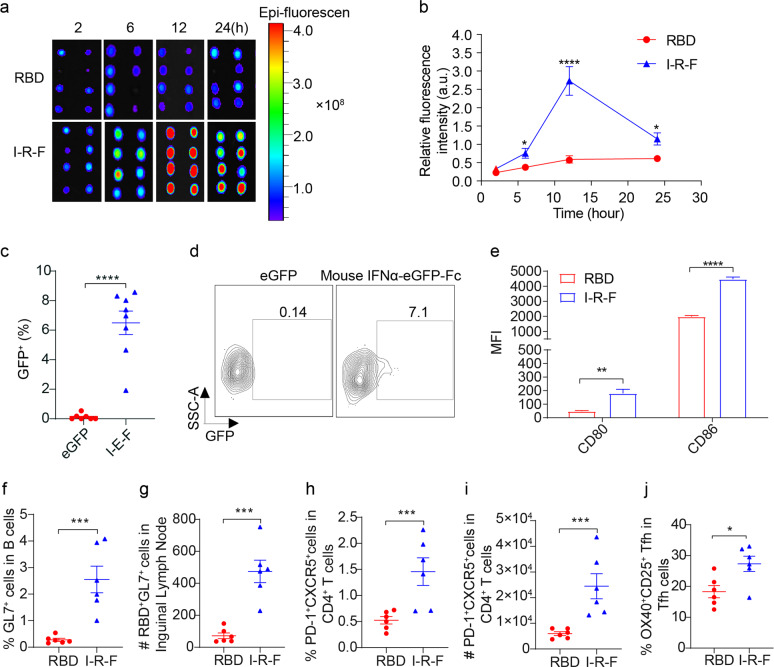
I-R-F efficiently stimulates Tfh and GC generation via targeting DC in lymph node. **a**, **b** BALB/c mice (*n* = 4/group) were subcutaneously injected Cy-5.5-labeled I-R-F or RBD at the tail base, and the mice were subjected to monitor the accumulation of labeled proteins in the inguinal lymph node at the indicated time points (**a**) and the fluorescence intensity of the DLN was quantified using the live imaging software (**b**). **c** C57BL/6 mice (*n* = 7 or 8/group) were i.m. injected 1 nmole of I-E-F (mouse IFNα-eGFP-Fc) or eGFP. 4 h after injection, mice were sacrificed. Lymphocytes from mouse iLNs were collected to analyze the capture of I-E-F or eGFP. The eGFP^+^ DCs (B220^−^CD11c^hi^MHC-II^+^) were determined by flow cytometry. The proportions of eGFP^+^ cells were analyzed. **d** The representative flow cytometry contour plots of eGFP^+^ DCs. **e** C57BL/6 mice (*n* = 5/group) were i.m. immunized with 10 μg of mouse IFNα-RBD-Fc (I-R-F) or equimolar RBD, and sacrificed 24 h later. DCs from iLNs were collected and analyzed with the maturation markers of CD80 and CD86 by flow cytometry. **f**–**j** C57BL/6 mice (*n* = 6/group) were i.m. vaccinated with I-R-F or RBD, and inguinal LN were collected 14 days after the initial immunization. **f** The proportion of GC cells (B220^+^CD3^−^GL7^+^CD38^+/−^) was determined. **g** The number of RBD^+^GL7^+^ B cells per inguinal lymph node was counted. **h** The frequency of Tfh (CD3^+^CD4^+^PD-1^+^CXCR5^+^) was detected in each group. **i** The number of Tfh (CD3^+^CD4^+^PD-1^+^CXCR5^+^) per inguinal LN was detected in each group. **j** The frequency of RBD-specific Tfh cells post stimulation with RBD peptide pool was measured by the proportion of CD25^+^OX40^+^ Tfh cells. Data are shown as mean ± SEM. *P* values in (**b**–**d**) were analyzed with the Student’s unpaired *t* test. *P* values in **e** and **f** were calculated by one-way ANOVA with multiple comparison tests. *P < 0.05, ***P < 0.001, ****P < 0.0001.

We hypothesized that the more robust immune responses induced by I-R-F than RBD might be attributed to the fused dimerized Fc, which improves draining to LNs. To evaluate this hypothesis, we labeled the RBD and I-R-F protein with fluorescein. After subcutaneous injection at the tail base, bilateral inguinal LNs were isolated at different time points post injection. Significantly increased fluorescence intensity was observed in the I-R-F group from 6 h to 24 h post injection, with the maximum difference occurring at 12 h post injection (Fig. 4a, b). Fluorescein-labeled RBD failed to effectively reach the LNs, likely due to its smaller molecular size. Considering the different labeling efficiencies of different proteins, which may influence the quantitative analysis of fluorescent molecules, we replaced RBD with enhanced green fluorescent protein (eGFP) to generate an IFNα-eGFP-Fc fusion protein (I-E-F). To ensure consistency with the recommended immunization method, we i.m. injected equimolar amounts of eGFP or I-E-F into the lateral thigh of mice. We analyzed eGFP^+^ myeloid cells in isolated DLNs by flow cytometry. The percentages of eGFP-positive DCs (Fig. 4c, d) and macrophages (Supplementary information, Fig. S4a, b) in I-E-F-vaccinated mice were remarkably higher than those in the group vaccinated with eGFP alone. These data suggest that I-R-F likely targets LNs much more efficiently than RBD. To directly examine DC maturation, likely resulting from IFNα stimulation, CD80 and CD86 expression in the I-R-F group was determined by flow cytometry. Compared to the RBD group, the I-R-F group presented much higher levels of CD80 and CD86 on DCs (Fig. 4e; Supplementary information, Fig. S4c).

The effective generation of T follicular helper cells (Tfhs) can result from improved antigen presentation to CD4^+^ T cells. Tfhs in lymphatic tissues are essential for forming germinal centers (GCs) and for B cell differentiation.^32,33^ We also found an increased percentage and number of total GC B cells (Fig. 4f; Supplementary information, Fig. S4d) and RBD-specific GC B cells (Fig. 4g; Supplementary information, Fig. S4d) in the I-R-F group compared with those in the RBD group. Additionally, a higher percentage and more Tfh cells were detected in inguinal LNs in the I-R-F group than the RBD group (Fig. 4h, i; Supplementary information, Fig. S4e). The characterization of antigen-specific Tfhs is essential for determining the mechanistic basis of antibody responses. Furthermore, an assay of activation-induced markers (AIMs) was performed, which showed that more RBD-specific Tfhs were induced in the I-R-F group than in the RBD group (Fig. 4j; Supplementary information, Fig. S4f). Together, these data suggest that I-R-F can target and activate DCs in LNs more efficiently than RBD, leading to stronger Tfh and GC reactions.

### The pan DR epitope (Pan) further enhances the immunogenicity of the I-R-F vaccine

**Fig. 5:**
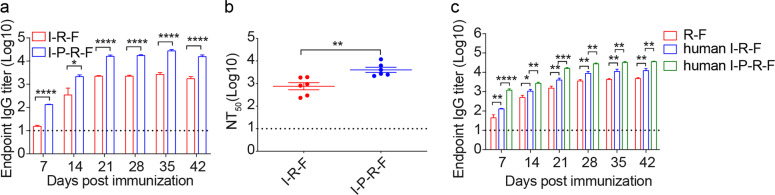
The pan DR epitope (Pan) further enhances the I-R-F immunogenicity. C57BL/6 mice (*n* = 6/group) were vaccinated i.m. with 0.1 μg of I-R-F or I-P-R-F using a two-dose immunization procedure. Sera were collected on days 7, 14, 21, 28, 35, 42 after the initial immunization. **a** RBD-specific IgG antibody titer was determined by ELISA. **b** The neutralization activity of vaccinated sera collected on day 28, as shown in **a**, was evaluated using a pseudovirus neutralization assay. **c** BALB/C mice (*n* = 8/group) were immunized i.m. with 1 μg of human IFNα-RBD-Fc (human I-R-F), human IFNα-Pan-RBD-Fc (human I-P-R-F), or equimolar RBD, respectively, and boosted with the same dose at a 14-day interval. Sera were collected on days 0, 14, 21, 28, 35, and 42 after initial immunization and analyzed by ELISA. The dashed line indicates the limit of detection. Data are shown as mean ± SEM. *P* values in **a** and **b** were analyzed with Student’s unpaired *t*-test. *P* values were calculated by one-way ANOVA with multiple comparisons tests in **c**. *P < 0.05, **P < 0.01, ***P < 0.001, ****P < 0.0001.

To avoid potential limitations and competition with RBD immunogenicity from dominant epitopes outside of the RBD in the S glycoprotein, we selected the RBD dimer as the only viral antigen. However, the RBD, a smaller polypeptide portion of the S protein, might contain a limited number of helper T cell epitopes for B cells and CTLs in a broader population. The pan DR-binding epitope (PADRE) is known to provide broad T cell responses by binding to common human HLA-DR types and mouse MHC molecules, IA^b^.^34,35^ To reduce the risk of limited helper epitopes for some HLA-DRs, we inserted PADRE into I-R-F and constructed the I-P-R-F vaccine to augment T cell responses (the schematic model is shown in Supplementary information, Fig. S5a). A clear peak of intact I-P-R-F fusion protein was visualized using size exclusion chromatography after a one-step protein-A column purification (Supplementary information, Fig. S5b). Additional SDS-PAGE also confirmed the purity of this fusion protein (Supplementary information, Fig. S5b). Real-time binding kinetics showed the high-affinity binding of I-R-F to hACE2, as determined by a BIAcore T100 system (Supplementary information, Fig. S5c). The bioactivity of IFNα in I-P-R-F was as high as that of the free IFNα molecule when measured by an antiviral infection biological assay (Supplementary information, Fig. S5d). The mice were vaccinated i.m. with a low dose of the indicated vaccines (0.1 μg). We observed an over 10-fold increase in antibody levels in mice immunized with I-P-R-F compared with those immunized with I-R-F (Fig. 5a). The neutralization activity in the antisera from vaccinated mice was also evaluated using a pseudovirus neutralization assay. Consistently, I-P-R-F resulted in up to 10-fold higher NAb levels in mice at a notably low dose (Fig. 5b). We also generated a human I-R-F and a human I-P-R-F with a site mutation (Q124R) on human IFNα for human IFNα to bind mouse IFNα receptor and thus allowed human IFNα to be functional in the mouse model^36^ (Supplementary information, Fig. S5b, c). Higher antibody titers in immunized mouse sera were induced by human IFNα-RBD-Fc than RBD-Fc, confirming the potency of human IFN in the mouse model. Furthermore, human IFNα-Pan-RBD-Fc triggered a much stronger antibody response to RBD than human IFNα-RBD-Fc, which confirmed the role of Pan in enhancing the immunogenicity of this poor antigen (Fig. 5c).

### I-P-R-F vaccination induces complete protection against a high-dose SARS-CoV-2 challenge in rhesus macaques

Rhesus macaques are a commonly used model for SARS-CoV-2 virology, pathology, and immunology studies and for screening antiviral vaccines and medicines. To investigate the immunogenicity of this newly designed vaccine in rhesus macaques, eight macaques were divided into four groups with a male and a female in each group and were i.m. immunized twice (on day 0 and day 14) with either 10 μg or 50 μg V0-1 (human I-P-R-F) with or without alum adjuvant. Impressively, all vaccinated groups generated very high levels of RBD-specific IgG antibodies, even with low-dose vaccination. High antibody titers were induced in macaques even by vaccines without adjuvants regardless of whether high or low vaccine doses were used (Supplementary information, Fig. S6a, b). Notably, a high titer of antiviral IgG has been maintained for 250 days thus far. The sera from vaccinated animals were subjected to neutralization assays with pseudovirus and live SARS-CoV-2 (Supplementary information, Fig. S6c, d). The high viral neutralization titers (the 50% neutralization titer (NT_50_) > 1000 and the focus reduction neutralization titer 50% (FRNT_50_) > 3000) indicate that this newly designed vaccine induced strong and long-lasting protective immunity.

**Fig. 6:**
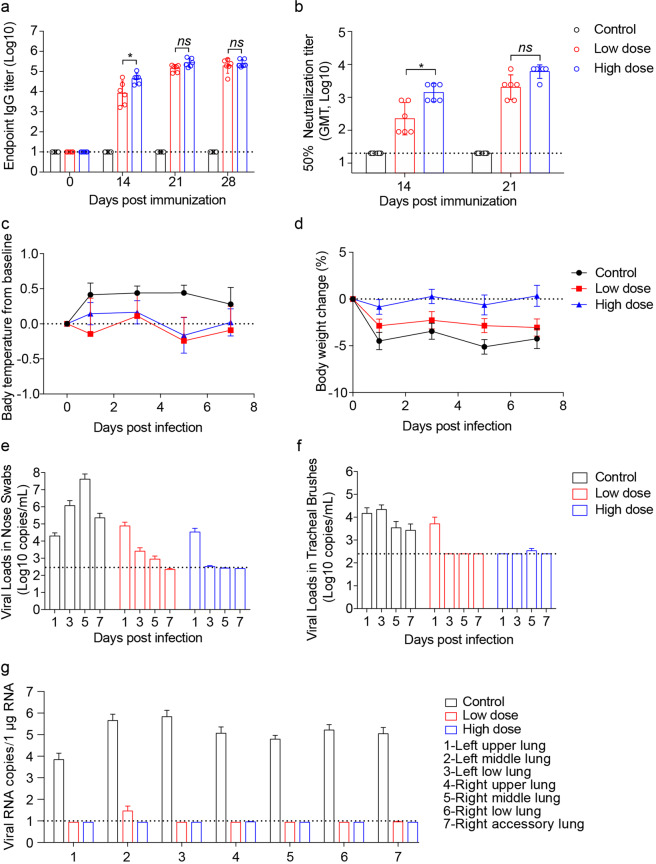
I-P-R-F vaccination induces complete protection against a high titer SARS-CoV-2 challenge in rhesus macaques. Rhesus macaques (*n* = 6/group) were administered twice with 10 μg or 50 μg of alum-adjuvanted I-P-R-F or alum alone as control via the i.m. injection and challenged with SARS-CoV-2 intranasally on day 21 after the initial immunization. Sera were collected on days 0, 14, 21 (before virus infection), and day 28 (after infection), and subjected to antibody detection. **a** The SARS-CoV-2-specific IgG was determined by ELISA. **b** Neutralization antibody titers were analyzed. **c**, **d** Body temperature (**c**) and body weight (**d**) were measured after the virus challenge. **e**, **f** Viral loads in nose swabs (**e**) and tracheal brushes (**f**) following the virus challenge were determined by qRT-PCR. **g** Viral load in various lung lobes of macaques challenged with SARS-CoV-2 on day 7 post infection. The dashed line indicates the limit of detection. Data are shown as mean ± SEM. *P* values were calculated by one-way ANOVA with multiple comparison tests. ns, not significant, *P < 0.05.

To further study whether the vaccine has a protective effect, eighteen rhesus macaques were divided into three groups (6 per group) and vaccinated twice (days 0 and 14) with 10 μg or 50 μg alum-adjuvanted V0-1 or alum alone as a control. All immunizations were administered via an i.m. injection in the lateral thigh. Macaques were intranasally challenged with a high dose of SARS-CoV-2 (1 × 10^7^ TCID_50_) 21 days after the initial immunization. We observed very high antibody titers against RBD in both the low- and high-dose vaccination groups, as determined by ELISA (Fig. 6a). Notably, high titers of NAbs were present in both the low- and high-dose vaccination groups, as determined by pseudovirus and live virus neutralization assays (Fig. 6b; Supplementary information, Fig. S6e). Although disease symptoms for SARS-CoV-2 infection are rather mild in rhesus macaques, when monkeys were infected with a high dose of virus, their body temperature was higher in the control group (Fig. 6c), as measured every two days after virus challenge. Similarly, lower body weight was observed in the control group but not in the immunized group (Fig. 6d).

Viral clearance in the upper respiratory tract in rhesus macaques has rarely been reported during the first seven days of infection, even after potent vaccination. However, our vaccine resulted in a significant reduction in viral loads in the nasal passages of macaques in the low-dose vaccine group and an undetectable viral load in the high-dose vaccine group only one day after high-titer virus infection, as determined by quantitative real-time reverse transcription-polymerase chain reaction (qRT-PCR) (Fig. 6e). Similarly, viral loads in tracheal brushes were also undetectable in both the low- and high-dose vaccination groups one day after virus infection (Fig. 6f). Viral loads in anal swabs of rhesus macaques were undetectable one day after infection in both the low- and high-dose vaccination groups (Supplementary information, Fig. S6f). For each group of vaccinated macaques, 84 specimens from lung lobes (14 samples/7 lobes/macaque) were collected on day 7 post infection and subjected to qRT-PCR to determine the viral loads in all areas of the lung lobes. A 4-log reduction in the average virus load was observed in the lungs of both the low- and high-dose vaccination groups, while extremely high viral loads were maintained in the lungs of control macaques (Fig. 6g; Supplementary information, Fig. 6g). These data demonstrate that V0-1 can generate highly effective protection against SARS-CoV-2 infection in both the upper and lower respiratory tracts, even with low-dose vaccination.

A randomized, double-blind, placebo-controlled phase I clinical trial was initiated in February 2021 to evaluate the safety and immunogenicity of our recombinant fusion protein COVID-19 vaccine (V-01) and was registered with chictr.org (ChiCTR2100045108). 180 healthy adults were recruited for the phase I study. Participants aged between 18 and 59 years or over 60 years (1:1) were sequentially enrolled and allocated into three subgroups (1:1:1) to receive vaccine (10, 25, or 50 μg) or placebo (V-01:placebo = 4:1) i.m. on days 0 and 21 with a sentinel and dose escalation design. Frequencies and percentages of adverse events were recorded. The primary serology outcomes included the seroconversion rate, geometric mean titers (GMTs), geometric mean fold increases (GMIs) of the RBD-binding antibody, and SARS-CoV-2 NAbs. There were no vaccine-related grade 3 adverse events (AEs) or serious AEs (SAEs) that were related to the investigational vaccine. All vaccinated adults acquired positive antibodies to RBD after vaccination with two doses of V-01. Clinical observation and analysis are ongoing.

## Discussion

Multiple types of SARS-CoV-2 vaccines have been developed and entered into a large number of clinical trials. The immunological effects and safety of these vaccines have been reported.^16,17,37–39^ An ideal vaccine should have the following properties: (1) efficacy and safety, as indicated by high-titer NAbs and T cell responses with no toxicity and antibody-dependent enhancement (ADE); (2) prolonged protective immunity and easy administration; and (3) “simplified” large-scale production, storage, and distribution. Here, we designed and evaluated a vaccine platform based on IFN-α-armed RBD, a dimerized human IgG1 Fc molecule (I-R-F). Supported by the results in mice and rhesus macaques, the I-R-F vaccine may be an effective prophylactic treatment for COVID-19 for the following reasons. (1) Potency: Due to its size, IFN-armed RBD dimerized by Fc primarily targets DLNs and increases antigen processing and presentation inside DLNs. Therefore, neutralizing RBD-specific IgG1 and IgG2a antibodies were induced by I-R-F at levels much higher than those in response to monomeric RBD. In addition, Th1, Th2, and CD8^+^ T cell responses were readily detected after vaccination. (2) Safety: RBD is the only exogenous antigen in the vaccine; thus, all antibodies induced target RBD to block viral entry. I-R-F elicited robust and long-lasting immune responses at a low dose (0.01 µg) and even without adjuvant. Unlike free IFN (18.7 KD), which is a smaller molecule, the I-R-F dimer has a much higher molecular weight (143 KD), which allows this molecule to enter DLNs to improve antigen presentation and DC maturation. The alum adjuvant used in this vaccine is well known and widely used without severe toxicity. Furthermore, alum-adjuvanted I-R-F allows the slow release of I-R-F molecules into DLNs, resulting in prolonged, effective, and safe immune challenges inside DLNs. On the other hand, there were no detectable autoimmune B or T cell responses to IFN. (3) Durability: The level of NAbs induced by the I-R-F vaccine was maintained for more than 250 days in the rhesus macaque model. (4) Accessibility: Fusion with Fc is easy in large-scale manufacturing and purification. Highly productive clones (one-gram level per liter) for large-scale human I-P-R-F (V-01) production have been confirmed in good manufacturing practice (GMP) grade manufacturing. (5) Portability: This fusion protein is stable at 4–25 °C for months, which notably allows for easier transport and storage in developing countries.

ADE induced by antibodies with weak neutralizing activities is an issue that should be considered when developing a SARS-CoV-2 vaccine.^40^ COVID-19 patients with higher antibody titers against SARS-CoV-2 may be associated with more severe illness, although the mechanism has not been fully determined.^41^ Subunit vaccines have been proposed that focus on S or RBD protein to induce NAbs, which could avoid producing ineffective antibodies and reduce ADEs. However, monomeric RBD is a weak immunogen, and multiple immunizations are needed to acquire protective immunity.^11^ To enhance immunogenicity, either modified S or RBD proteins, such as S trimer and RBD dimer proteins, were developed, which help to mimic the native 3D conformation of oligomerized S protein in vivo^5,42^ or form protein nanoparticles by displaying the RBD.^43,44^ However, the large-scale production of these new adjuvants remains challenging. Additionally, RBD fused with the Fc domain could be a better choice than a simple RBD or S protein.^7–9^ At equimolar levels, both I-R-F and I-P-R-F induced stronger humoral immunity than RBD dimer and RBD-Fc. In general, protein vaccines do not have enough protective immunogenicity without the use of additional potent adjuvants. IFN can provide an adjuvant effect without detectable toxicity while enhancing immunogenicity and antigen presentation.

For protein vaccines, Th1 or Th2 responses are mainly dependent on the type of adjuvant. Notably, enhanced immunopathology was associated with a Th2-biased response.^10,45–47^ The choice of adjuvant is thus crucial. Alum is a universal adjuvant used with protein vaccines that leads to high antibody titers, but it preferentially induces a Th2-biased response.^48^ Moreover, protein-based vaccines may elicit weak or even no CD8^+^ T cell responses. All of these drawbacks prevent protein vaccines from being ideal and effective vaccine candidates. Herein, we designed an I-R-F vaccine with extremely strong immunogenicity even at extreme low doses or without adjuvant. Mechanistically, multiple factors contribute to such robust immune responses. (1) IFNα is the most potent cytokine for antigen processing and presentation by DCs.^49^ With I-R-F, we observed more efficient antigen uptake by DCs. (2) IFNα could also be used as an adjuvant to improve the generation of Tfhs.^50^ We found that the I-R-F vaccine induced higher percentages of Tfhs and GC B cells, as well as high RBD-specific IgG1 and IgG2a titers, indicating strong humoral immunity. Moreover, IFN might help Th1 and CTL responses even with alum. Indeed, the I-R-F vaccine shift the alum-induced Th2-biased response to a more robust Th1/Th2-balanced response. (3) Importantly, the Pan epitope further enhances the I-R-F vaccine-induced robust CD4^+^ and CD8^+^ T cell responses that contribute to eliminating virus-infected cells. (4) The additional Fc allows RBD to naturally form dimers and make I-R-F a much larger molecule than RBD, thus could reach DLNs more efficiently.

High doses and long-term use of various types of type I IFN have been approved for the infection of several virues, but some patients showed various toxicities.^51–53^ In preclinical studies, no significant body weight loss or abnormal ALT/AST above background levels was observed after I-R-F vaccination. There were no significant increases in cytokines induced by the vaccine at the tested doses. We did not observe any side effects in the I-R-F vaccinated host.

In light of the significant immunogenicity, complete protection in both the upper and lower respiratory tracts, and undetectable toxicity of I-P-R-F in macaques, this unique protein vaccine could potentially be used at very low dosages and as a single vaccine. Furthermore, this protein vaccine maintains its potency without adjuvant, which may allow intranasal vaccination in all age groups, especially young children. Nasal I-R-F vaccine investigations are underway because it triggers a mucosal IgA response to prevent the virus from invading mucosa of the nose and upper respiratory tract or infecting others. Armed-IFN, which overcomes the poor immunogenicity of some antigens, can be broadly applied in vaccines to prevent other infectious diseases during future pandemics. The enhanced immunogenicity due to this novel strategy is not limited to protein vaccines, but could also be applied to other vaccine forms, such as genetic or viral vector-based vaccines.

## Materials and methods

### Ethics statement

All mouse experiments were approved by the Biomedical Research Ethics Committee of the Institute of Biophysics of the Chinese Academy of Sciences (CAS) and were performed in compliance with the Guidelines for the Care and Use of Laboratory Animals of the Institute of Biophysics. Non-human primates, rhesus macaques, immunogenicity studies were performed in the animal facility of Guangxi Fangchenggang Biotechnology Development Co., Ltd. (GFBDCL), according to the guidelines of the Committee on Animals of GFBDCL (approval No. SYXK2018-0004/200005). Non-human primates, rhesus macaque, infection studies were performed in the Biosafety Level 3 (BSL-3) in the Kunming National High-Level Biosafety Research Center for Non-Human Primates, Center for Biosafety Mega-Science, Kunming Institute of Zoology (KIZ), CAS, according to the guidelines of the Committee on Animals of KIZ, CAS (approval No. IACUC20005).

### Animals

Female (6–8-week-old) BALB/c mice and C57BL/6 J mice were obtained from Vital River (Beijing) and bred under specific pathogen-free (SPF) conditions in the animal facility of the Institute of Biophysics and the Institute of Microbiology, CAS. Eight healthy rhesus macaques were used for vaccine immunogenicity analysis. These macaques (4 male, 4 female, between 2.5–4-year old) were purchased from Guangxi Fangchenggang Biotechnology Development Co., Ltd. and housed in a clean-level animal facility of Guangxi Fangchenggang Biotechnology Development Co., Ltd. Additional eighteen healthy rhesus macaques were used for virus infection experiment. These healthy ChRMs (male *n* = 9, female *n* = 9, 3–5 years old) were sourced from the Kunming Primate Research Center, KIZ, CAS, and housed in the Kunming National High-Level Biosafety Research Center for Non-Human Primates, Center for Biosafety Mega-Science, KIZ, CAS. All animals recruited in this study are healthy and not involved in other studies.

### Cell lines, virus, and reagents

293 F cells (Gibco) were maintained in SMM-TII medium (M293TII, Sino Biological), incubated in Polycarbonate Erlenmeyer Flasks under 135 rpm speed in an orbital shaker and cultured in an 8% incubator at 37 °C. Vero E6 cells were obtained from ATCC, and 293-ACE2 was kindly provided by Prof. Zheng Zhang (National Clinical Research Center for Infectious Disease, Shenzhen Third People’s Hospital, Shenzhen, Guangdong, China). Cells were cultured in 5% CO_2_ and maintained in Dulbecco’s modified Eagle’s medium (DMEM) supplemented with 10% heat-inactivated fetal bovine serum (FBS), 100 U/mL penicillin, and 100 mg/mL streptomycin.

SARS-CoV-2 pseudovirus was produced in house as previously described.^8^ Briefly, human immunodeficiency virus backbones expressing firefly luciferase (pNL43R-E-luciferase) and pcDNA3.1 (Invitrogen) expression vectors encoding the SARS-VoV-2 S protein were co-transfected into 293 T cells (ATCC). Viral supernatants were collected 48 h later. Viral titers were measured as luciferase activity in relative light units (Bright-Glo Luciferase Assay Vector System, Promega Biosciences).

The SARS-CoV-2 strain (Bata/Shenzhen/SZTH-003/2020, EPI_ISL_406594 at GISAID) was obtained from a nasopharyngeal swab of an infected patient, and the virus was stock propagated in Vero-E6 cells.

The SARS-CoV-2 strain 107 (NMDC000HUI) was used in rhesus monkey infection. This virus strain was obtained from Guangdong Provincial Center for Disease Control and Prevention, Guangdong, China. The virus was stock and amplified in Vero-E6 cells.

His-tagged hACE2 protein, rabbit anti-SARS-CoV-2 nucleocapsid, and HRP-conjugated goat anti-rabbit IgG (H + L) antibody antibodies were purchased from Sino Biological Inc. (Beijing, China). The peptide pool spanning the SARS-CoV-2 RBD consisting of 53 peptides (15-mer) overlapping by 11 amino acids were synthesized by China Peptides Co., Ltd (Shanghai, China).

### Protein expression and purification

The COVID-19 vaccine protein was expressed in 293 F cells, as described previously.^22,54^ The coding sequence for SARS-CoV-2 RBD spanning S protein 319–541 (GenBank: YP_009724390) was codon-optimized for mammalian cells and synthesized by GENEWIZ, China. For I-R-F expression, murine IFNα4 was fused to the N-terminus of RBD with a (G4S)_4_ linker. The IFNα-RBD sequence was then cloned into the PEE12.4 (Lonza) with a human IgG1 Fc, forming the IFNα-RBD-Fc (I-P-F) fusion protein. The plasmid was transiently transfected into 293 F cells. The supernatant was collected seven days after transfection, and the protein within the supernatant was purified with a Protein A-Sepharose column (GE Healthcare) according to the manufacturer’s instruction for primary purification. Then, the eluted protein was further purified using a Superdex 200 Increase 10/300 GL column (GE Healthcare). The purity and size of the protein were analyzed by sulfate-polyacryl-amide gel electrophoresis (SDS-PAGE). R-F, I-E-F, I-P-R-F with a CD4 helper epitope (PADER), I-R-F (human) with a human IFNα2 substituting for murine IFNα4 and I-P-R-F (human) were expressed and purified with the same method as described above.

Recombinant SARS-CoV-2 RBD protein (rRBD) was also expressed in 293 F cells. In brief, the coding sequence for RBD with a 6× His tag on C terminus was cloned into the pEE12.4 vector without human IgG1 Fc. The plasmid was transiently transfected into 293 F cells. The supernatant was harvest on day 7, and the protein was purified using Ni-NTA agarose beads (GE Healthcare). The protein was further purified on a Superdex 200 Increase 10/300 GL size exclusion column (GE Healthcare). SDS-PAGE was performed to determine the purity and size of the protein. The eGFP and dimer-RBD protein were expressed and purified with the same method as rRBD.

### Surface plasmon resonance (SPR) analysis

SPR assays were performed by a BIAcore T100 instrument with a CM5 sensor chip (GE Healthcare). Experiments were carried out at 25 °C in binding buffer (PBS, 0.05% Tween 20, pH 7.4). The CM5 sensor chip was used to capture about 100 response units (RUs) RBD of SARS-CoV-2, RBD-Fc, mouse IFNα-RBD-Fc, mouse IFNα-Pan-RBD-Fc, human IFNα-RBD-Fc and human IFNα-Pan-RBD-Fc for 3 min, respectively. A two-fold serial dilution of hACE2 (from 6.25 nM to 200 nM) was run across the chip surface with a flow rate of 30 μL/min, and the real-time response was recorded. The resulting data were analyzed by global fitting to a 1:1 binding model with Biacore Evaluation Software.

### The anti-viral activity of IFNα

The IFNα bioactivity was determined by the anti-viral infection assay using the L929 fibroblast cell line sensitive to VSV infection. L929 cells were seeded in 24-well plates (4 × 10^5^ per well) and incubated for approximately 16 h. A serial dilutions of I-R-F and I-P-R-F was incubated into the medium of L929 cells and incubated for 24 h at 37 °C with 37% CO_2_. The cells were infected with VSV-GFP virus (MOI = 5) and further cultured for 30 h. Cells were collected and fixed by 4% PFA and subjected to analysis using a FACS Fortessa flow cytometer (BD Bioscience). Cells with GFP signal positive were considered as the virus-infected cells.

### Mouse vaccination

The immunogen used to immunize mice was diluted with PBS and mixed with or without a fixed-dose (20 ug per mouse) of alum adjuvant (SEVA, Germany). To make alum adsorb the immunogen efficiently, the mixture was kept rolling overnight at 4 °C. Female (6–8-week-old) BALB/c or C57BL/6 mice were immunized i.m. or subcutaneously with different immunogens in 100 μL using insulin syringes. PBS containing alum was used as a control. Serum samples were collected at indicated time points to determine the levels of SARS-CoV-2 RBD-specific IgG and neutralization antibody. The details of mouse vaccination were described in the figure legends.

### ELISA

The 96-well plates (Conning, USA) were coated with 100 uL SARS-Cov-2 RBD (1.5 ug/mL) overnight at 4 °C. Plates were washed with PBS and blocked with a blocking buffer (PBS containing 5% fetal bovine serum, FBS) on the next day. Immunized animal serum samples were serially diluted and added to the blocked plates, followed by incubation at 37 °C for 1 h. Plates were then washed with PBST (PBS containing 0.05% Tween 20) and incubated with goat anti-mouse IgG-HRP (1:5000, Cwbiotech) or goat anti-monkey IgG-HRP (1:10,000, Invitrogen) at 37 °C for 30 min. To detect the Ig subclasses, goat anti-mouse IgG1 (1:5000, Proteintech), goat anti-mouse IgG2a (1:5000, Proteintech) was added. Plates were washed with PBST, and HRP substrate TMB was added. The reactions were stopped by 2 M sulfuric acid. The absorbance at 450–630 was read using a microplate reader (Molecular Devices). The endpoint titers were defined as the reciprocal of max serum dilution at which the absorbance was higher than 2.5-fold of the background.

### Pseudovirus neutralization assay

The pseudovirus neutralization was carried out as described previously. In brief, the pseudovirus was produced by co-transfection of the plasmid expressing firefly luciferase (pNL43R-E-luciferase) and pcDNA3.1 expressing the SARS-CoV-2 S protein into 293 T cells. After 48 h, the viral supernatant was collected, and viral titers were determined by luciferase activity in relative light units. To evaluate the neutralizing activity of vaccinated mice serum, 293-hACE2 cells were seeded into 96-well plates (2 × 10^4^ per well) and 3-fold serially diluted heat-inactivated serum samples were incubated with 100 TCID_50_ of pseudovirus for 1 h at 37 °C. Medium mixed with pseudovirus was used as control. The mixture was transferred to the 96-well plates, and the plates were continued to incubate for another 24 h. According to the manufacture’s instruction, the luciferase substrate was added, and luciferase activity was determined by the Bright-Lite^TM^ Luciferase Assay System (Vazyme). The NT_50_ was defined as the reciprocal of serum dilution at which the relative light units (RUL) were reduced by 50% compared with virus control wells.

### FRNT

Vero-E6 cells were seed into 96-well plates with a density of 2 × 10^4^ per well. Sera from immunized animals and convalescent COVID-19 patients were serially diluted and mixed with 75 μL of authentic SARS-CoV-2 (8 × 10^3^ focus-forming units (FFU)/mL). The mixture was incubated for 1 h at 37 °C and then transferred to the 96-well plates seeded with Vero E6 cells. Plates were incubated for 1 h at 37 °C. The inoculums were removed, and the plates were overlaid with medium (100 μL DMEM containing 1.6% carboxymethylcellulose (CMC)). The plates were incubated for another 24 h at 37 °C. Then, the supernatant was removed, and cells were fixed with 4% Paraformaldehyde for 30 min. Cells were subsequently permeabilized with PBS containing 0.2% Triton X-100. After washed with PBS for three times, the cells were incubated with cross-reactive rabbit anti-SARS-CoV-2 nucleocapsid IgG (Sino Biological) for 1 h at 37 °C. After incubated with the primary antibody, plates were washed three times with PBST followed by the incubation of HRP-conjugated goat anti-rabbit IgG (Jackson ImmunoReseach). Cells were further incubated for 1 h at 37 °C. After washing, the KPL TrueBlue peroxidase substrates (Seracare Life Science) were added to the plates. The supernatant was removed, and the plates were washed three times with deionized water 5 min later. The numbers of SARS-CoV-2 foci were read using an ELISPOT reader (Cellular Technology). The FRNT_50_ was defined as the sera dilution at which neutralization antibodies inhibited 50% of the viral infection.

### Authentic SARS-CoV-2 neutralization CPE assay

Vero-E6 cells were harvested and seeded into 96-wells plates with 2 × 10^4^ cells per well and cultured at 37 °C overnight. The serum samples from immunized rhesus macaques were inactivated at 56 °C for 30 min and serially diluted with cell culture in 3-fold steps. The diluted serum samples were mixed with a virus suspension containing 100 TCID_50_ authentic SARS-CoV-2 in an equal volume. After neutralization in a 37 °C incubator for 1 h, the mixtures were transferred to the 96-well plates containing Vero-E6 cells. Inoculated plates were cultured in a CO_2_ incubator at 37 °C for 6 days. Cytopathic effect (CPE) of each well was scored, and the neutralization titer was calculated as the reciprocal of serum dilution at which neutralization antibodies inhibited 50% of viral infection.

### ELISPOT assay

Murine IFNγ and IL-4 ELISPOT assays were carried out according to the manufacture’s protocols for mouse IFNγ and IL-4 ELISPOT kit (BD Bioscience). Splenocytes from immunized mice were seeded in the plates with a density of 2 × 10^5^ cells per well and incubated with the peptide pool of 15-mer peptides with 11 overlapping amino acids for SARS-CoV-2 RBD protein (5 μg/mL) in pre-coated 96-well ELISPOT plates with anti-mouse IFNγ or anti-mouse IL-4 antibody for 48 h at 37 °C., Concanavalin A (ConA, Sigma) was used as a positive control or medium as a negative control. Then, the cells were removed, and biotinylated anti-mouse IFNγ or IL-4 detection antibody (BD Bioscience) was added to the plates, followed by incubation for 2 h at room temperature. The plates were washed three times with PBST before adding Streptavidin-HRP (BD Bioscience). The BD ELISPOT AEC substrate (BD Bioscience) was used to develop the spots. Spots were counted and analyzed using an automated ELOSPOT reader (Cellular Technology). For the B-cell ELISPOT assay, splenocytes from immunized mice were seeded in the plate coating with 2.5 μg/mL RBD protein. Cells were removed 16 h post incubation, and biotinylated goat anti-mouse IgG (BBI life sciences) was added into plates, followed by incubation for 2 h at room temperature. The plates were washed three times with PBST before adding Streptavidin-HRP (BD Bioscience). The BD ELISPOT AEC substrate (BD Bioscience) was used to develop the spots. Spots were counted and analyzed using an automated ELOSPOT reader (Cellular Technology).

### Intracellular cytokine staining (ICCS)

To evaluate the cytokine expression in antigen-specific T cells, ICCS assay was performed. Mouse splenocytes were seeded in U-bottom 96-well plates (1 × 10^6^/well) and stimulated with a peptide pool for SARS-CoV-2 RBD protein as described above. After 12-h stimulation, the cells were then incubated with 5 μg/mL Brefeldin A (Biolegend) for another 6 h, Then, the cells were collected and stained with anti-CD3 (Biolegend), anti-CD4 (Biolegend), anti-CD8 (Biolegend), and the LIVE/DEADTM dyes. Subsequently, the cells were fixed and permeabilized using a fixation/permeabilization kit (BD bioscience), and finally performed with intracellular staining for IFNγ (XMG1.2), IL-2 (JES6-5H4), TNFα (MP6XT22), IL-4 (11B11). All data were acquired using a FACS Fortessa flow cytometer (BD Bioscience) and analyzed with Flowjo software (TreeStar).

### In vivo imaging analysis

BALB/c mice were subcutaneously injected with 0.1 μmoL (cy5.5) of different cy5.5-labeled proteins (RBD, I-R-F) at the tail base. The inguinal LNs were extracted for imaging DLN and were imaged at indicated time points post injection using an in vivo imaging system (IVIS) Spectrum (PerkinElmer). The fluorescence imaging data were analyzed by Living Image software (PerkinElmer).

### Flow cytometry analysis

The DLNs were excised and digested into a single-cell suspension. 2 × 10^6^ cells were blocked with anti-CD16/32 (anti-FcγIII/II receptor, clone 2.4G2) and stained with specific fluorescence-labeled antibodies. For evaluating antigen-presenting cell maturation in immunized mice, DCs were incubated with anti-CD11c (N418) and anti-MHCII (M5/114.15.2). For phenotypic maturation analysis, anti-CD80 (16-10A1) and anti-CD86 (GL-1) were used. For Tfh and GC B cell analysis, cells in DLN were stained with anti-CD3 (17A2), anti-CD4 (RM4-5), anti-CD8 (53-6.7) anti-PD1 (RMP-30), anti-CXCR5 (L138D7), anti-B220 (RA3-6B2), anti-GL-7 (GL7), anti-CD95 (SA367H8). For in vivo uptake assay, the cells were labeled with anti-CD11c (N418), anti-MHCII (M5/114.15.2), anti-CD11b (M1/70) and anti-F4/80 (BM8). All the samples were acquired by BD LSRFortessa flow cytometer (BD Bioscience), and the data were analyzed with Flowjo software (TreeStar).

### Immunogenicity of I-P-R-F in rhesus macaques

To evaluate the immunogenicity of I-P-R-F in non-human primates, a total of 8 rhesus macaques (4 male and 4 female, weighing 3–5 kg) purchased from Guangxi Fangchenggang Biotechnology Development Co., Ltd. were randomly assigned into four groups with one male and one female in each group and i.m. immunized with high does (50 μg) or low dose (10 μg) of human I-P-R-F (V0-1) with or without alum as adjuvant two times at a 14-day interval. Serum samples were collected at indicated time points, and the SARS-CoV-2-specific IgG and neutralization antibody titers in serum were determined.

### SARS-CoV-2 challenge in rhesus macaques

A total of 18 Rhesus macaques (3–5-year old) were recruited and assigned into three groups. Macaques in group 1 were i.m. immunized with PBS formulated with the alum-adjuvant as control. Group 2 and Group 3 were vaccinated with 50 μg (high dose) or 10 μg (low dose) of I-P-R-F (V0-1), respectively. Animals received a two-dose immunization procedure on day 0 and day 14 after the initial vaccination. The macaques were intranasally challenged with 10^7^ TCID_50_ of SARS-CoV-2 on day 7 after the second immunization. Animal body temperature and body weight were recorded after the virus challenge. Serum samples were collected on days 0, 14, 21, and 28 after vaccination and subjected to antibody assays. The viral loads in nose swabs, tracheal brushes, and anal swabs were determined by quantitative reverse transcription-polymerase chain reaction (qRT-PCR) at indicated time points, and the lung tissues were collected on day 7 post infection and used to determine the viral load.

### qRT-PCR

Viral RNA in swabs, tracheal brushes, and lung tissues was determined by qRT-PCR. In brief, total RNA in swabs and tracheal brushes was extracted using the QIAamp viral RNA Mini kit (QIAGEN) according to the manufacturer’s instructions. Lung tissues were homogenized, and RNA was extracted with an RNeasy Mini kit (QIAGEN). The viral RNA copies were determined using THUNDERBIRD^TM^ probe one-step qRT-PCR kit (TOYOBO) with the following primers and probes: forward primer 5’-GGGGAACTTCTCCTGCTAGAAT-3’, reverse primer 5’-CAGACATTTTGCTCTCAAGCTG-3’, and probe FAM-TTGCTGCTGCTTGACAGATT-TAMRA-3’. SARS-CoV-2 RNA reference standard (National Institute of metrology, china) was serially diluted and performed to generate the standard curve.

### Statistical analysis

All statistical analyses were performed using Graphpad Prism 8.0. Data are shown as the mean ± SEM. An unpaired Student’s two-tailed *t*-test was used to determine statistical significance for comparison between two groups. One-way ANOVA with Turkey’s multiple comparison test or two-way ANOVA with Turkey’s multiple comparisons test was conducted to compare differences among multiple groups. *P* values of <0.05 were considered significant. *P < 0.05, **P < 0.01, ***P < 0.001 and ****P < 0.0001, ns, no significance.

## Supplementary information

Supplementary information, Fig. S1

Supplementary information, Fig. S2

Supplementary information, Fig. S3

Supplementary information, Fig. S4

Supplementary information, Fig. S5

Supplementary information, Fig. S6
