# Supplementary material for: Interferon-armed RBD dimer enhances the immunogenicity of RBD for sterilizing immunity against SARS-CoV-2
Source: Cell Res. 2021 Jul 15;31(9):1011–23. doi: 10.1038/s41422-021-00531-8 (PMC8280646; doi:10.1038/s41422-021-00531-8)

Supplementary information, Fig. S1. I-R-F induces more robust neutralization antibodies than RBD. (a) BALB/c mice (n=6/group) were i.m vaccinated with 10 μg of I-P-F or equimolar of RBD, RBD-dimer, and R-F protein or PBS control with a single vaccination regimen. The levels of RBD-specific IgG in serum on day 28 after immunization were determined by ELISA. **(b)** BALB/c Mice (n=6/group) were intramuscularly vaccinated with 1 μg of I-R-F or equimolar R-F plus IFNα protein without adjuvant and boosted with the same dose at a 14-day interval. Serum samples were collected on day 14 after the second immunization to evaluate the levels of RBD-specific IgG. **(c)** BALB/c mice (n=8/group) were immunized intramuscularly or subcutaneously with alum-adjuvanted 10μg of I-R-F and boosted on day 14 after initial immunization with equivalent dose. Serum samples were collected every week to determine the SARS-CoV-2-specific IgG antibody titers by ELISA. **(d)** BALB/c mice (n=8/group) were immunized intramuscularly with alum-adjuvanted 10 μg of I-R-F by using a single dose (day 0), two-dose (day 0/14), and three-dose (day 0/14/28) immunization procedures, respectively. Sera were collected on days 7, 14, 21, 28, 35, and 42 after the initial immunization and analyzed by ELISA to determine the IgG titer. **(e)** The neutralization antibody titers in serum described in Fig (2d-2f) on day 28 were determined by SARS-CoV-2 pseudovirus neutralization assay. The dashed line indicates the limit of detection. Data are shown as mean ± SEM. *P*-values were calculated by one-way ANOVA with multiple comparison tests. ns (not significant), *$P < 0.05$, **$P < 0.01$, ***$P < 0.001$, ****$P < 0.0001$.

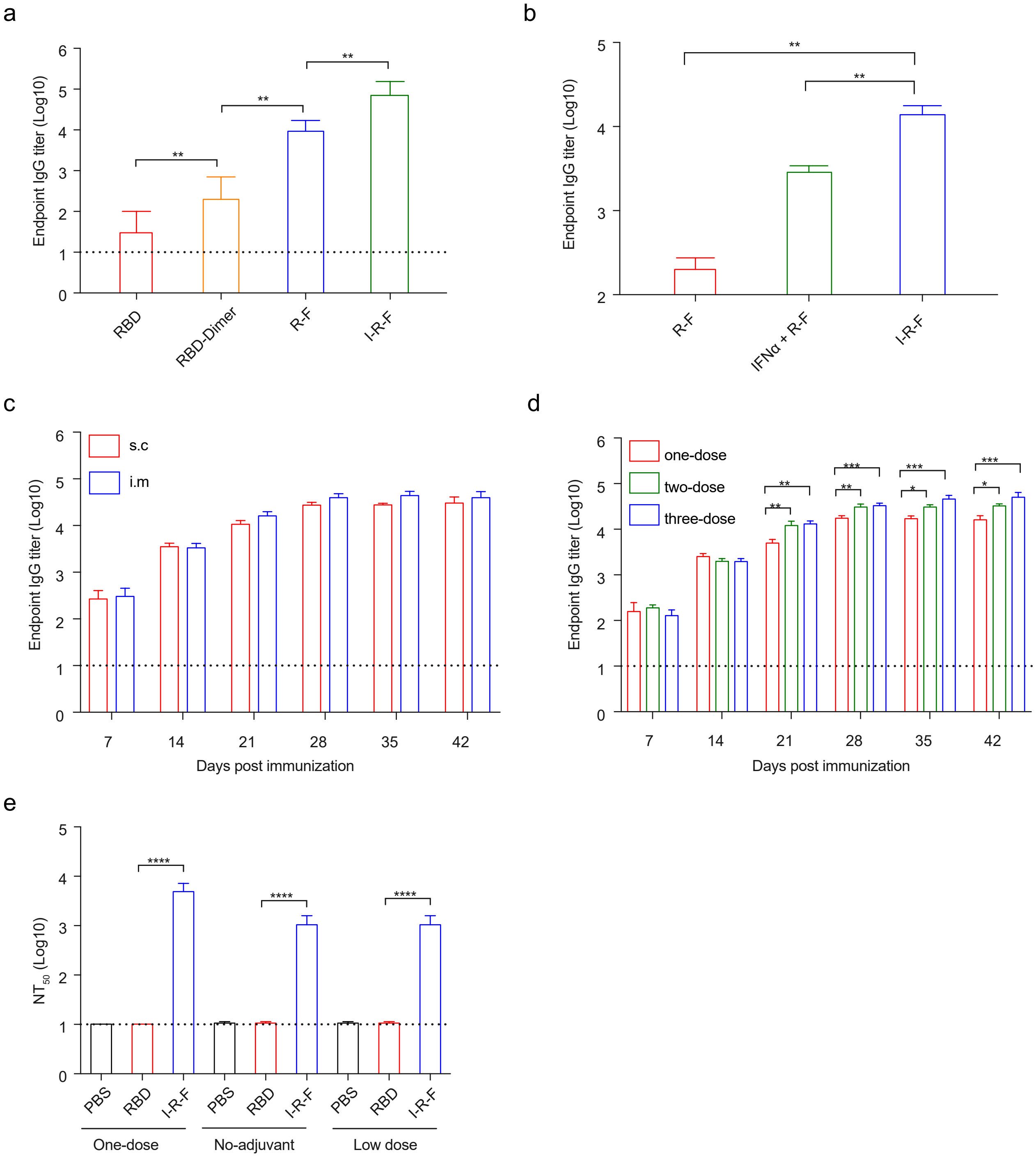

Supplement: Supplementary file 1 — Supplementary information, Fig. S1 [file 41422_2021_531_MOESM1_ESM.pdf]
