# Supplementary material for: Interferon-armed RBD dimer enhances the immunogenicity of RBD for sterilizing immunity against SARS-CoV-2
Source: Cell Res. 2021 Jul 15;31(9):1011–23. doi: 10.1038/s41422-021-00531-8 (PMC8280646; doi:10.1038/s41422-021-00531-8)

Supplementary information, Fig. S2. Safety evaluation of I-P-F in mice. (a-c) BALB/c mice (n = 6) were intramuscularly immunized with 10 μg, 50 μg or 100ug of I-R-F and the body weight, serum cytokines and biochemical indexes were determined. **(a)** Body weight changes were monitored and analyzed. **(b)** Serum inflammatory cytokines were determined by Cytometric Beads Array (CBA). **(c)** ALT and AST levels were analyzed by an automatic biochemical analyzer. The dotted lines indicate the upper limit of the normal range of ALT or AST using mean ± 2×SD of PBS group. **(d)** Evaluation of the IFNα specific antibody levels by ELISA. BALB/c Mice (n = 6/group) were intramuscularly vaccinated with 10 μg of I-R-F or equimolar RBD protein and boosted with the same dose at a 14-day interval. PBS was performed as a negative control. Sera were collected on day 28 after the initial immunization and subjected to determine the anti-murine IFNα level. The dashed line indicates the limit of detection. Data are shown as mean ± SEM. *P*-values were calculated by one-way ANOVA with multiple comparison tests. ns (not significant), **P* < 0.05, ***P* < 0.01, ****P* < 0.001, *****P* < 0.0001.

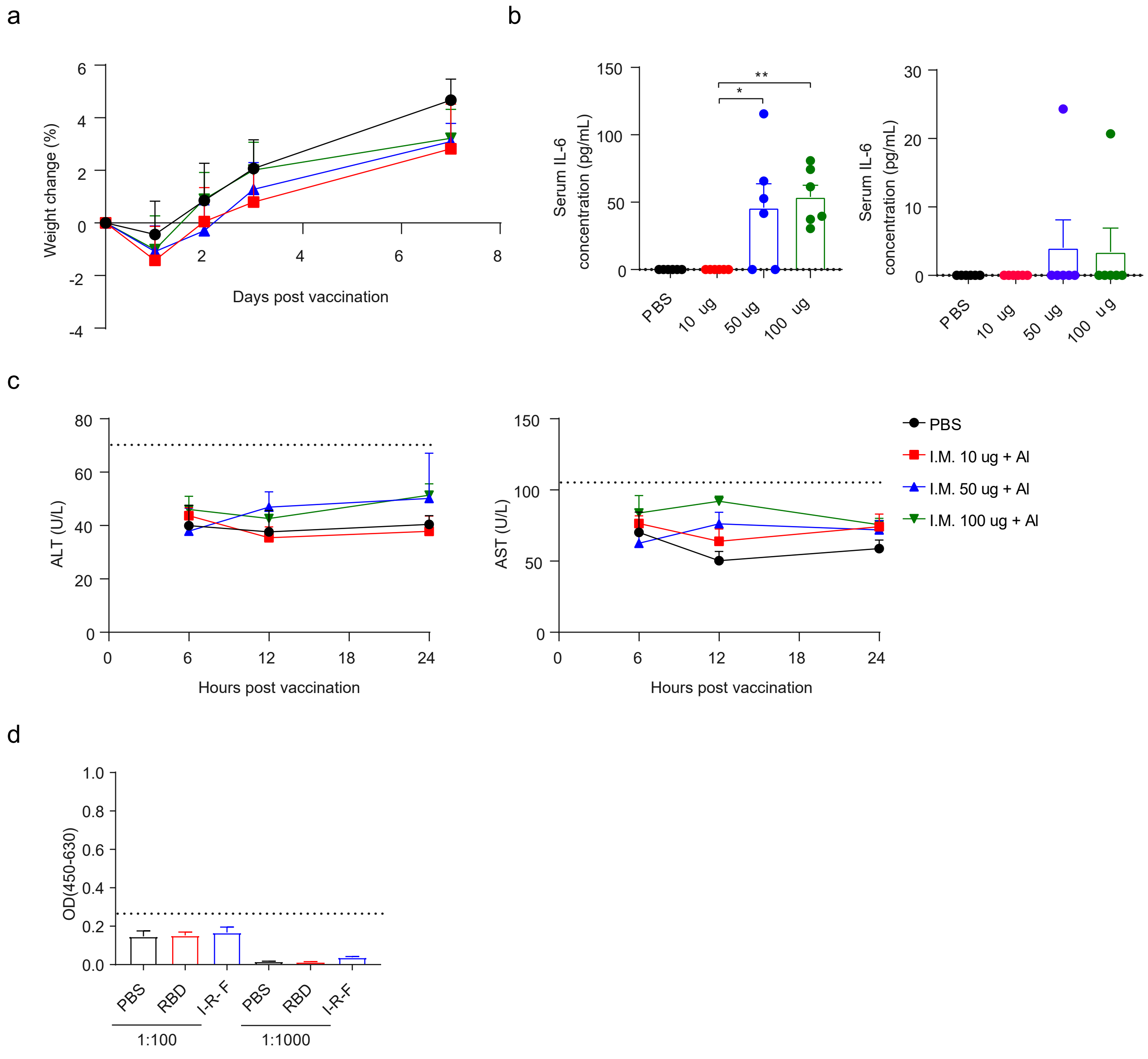

Supplement: Supplementary file 2 — Supplementary information, Fig. S2 [file 41422_2021_531_MOESM2_ESM.pdf]
