# Supplementary material for: Interferon-armed RBD dimer enhances the immunogenicity of RBD for sterilizing immunity against SARS-CoV-2
Source: Cell Res. 2021 Jul 15;31(9):1011–23. doi: 10.1038/s41422-021-00531-8 (PMC8280646; doi:10.1038/s41422-021-00531-8)

Supplementary information, Fig. S3. B and T cells immune response related to Figure 3. (a) Gating strategy for evaluating RBD-specific memory B cells in the spleen related to Fig. 3c was present. **(b-d)** The raw data for B cell ELISPOT, IFNγ, and IL-4 ELISPOT related to Fig. 3b, 3d and 3e were present. The representative flow cytometric contour plots related to Fig. 3f, and Fig. 3g were shown in **(e)** and **(f)**.

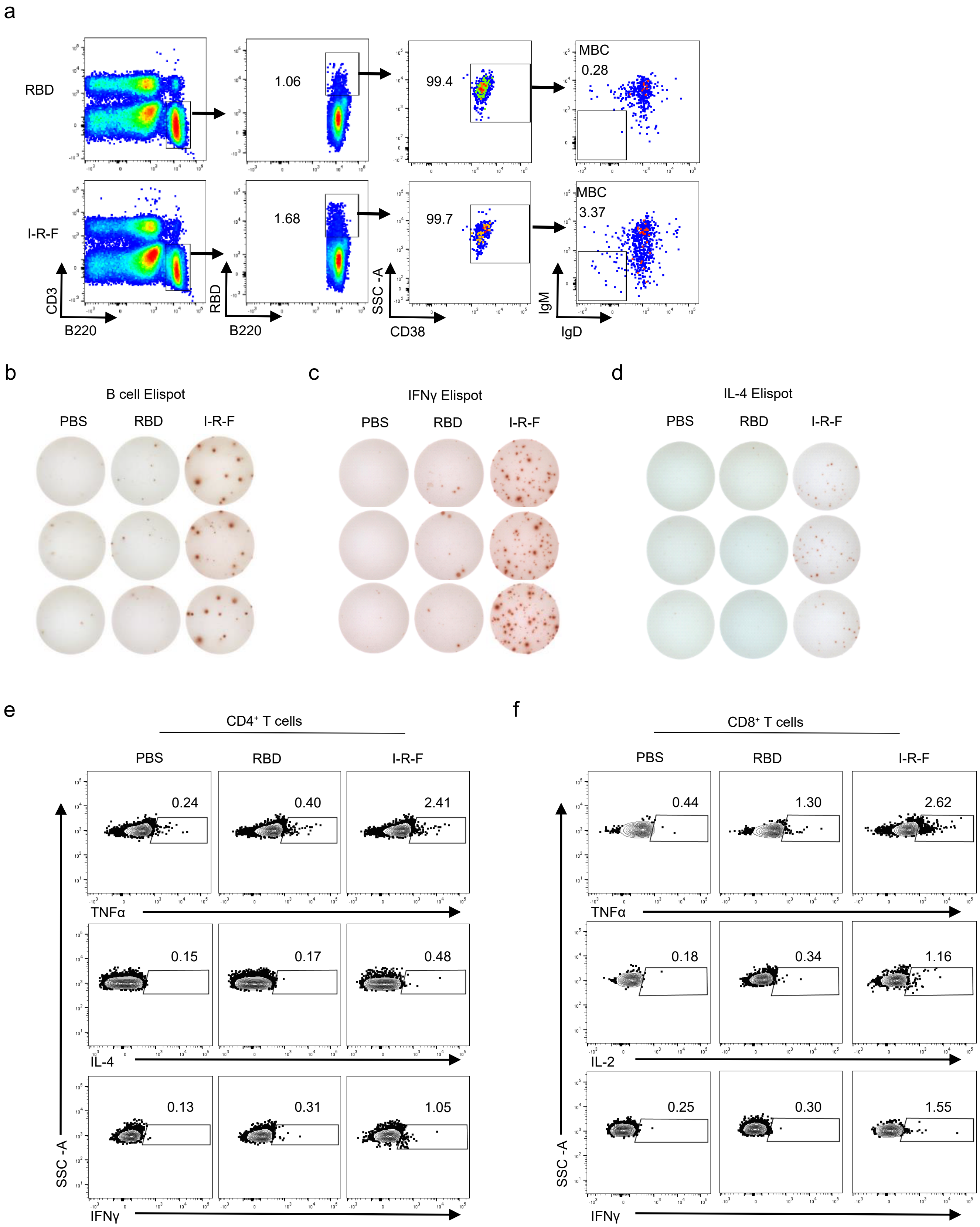

Supplement: Supplementary file 3 — Supplementary information, Fig. S3 [file 41422_2021_531_MOESM3_ESM.pdf]
