# Supplementary material for: Interferon-armed RBD dimer enhances the immunogenicity of RBD for sterilizing immunity against SARS-CoV-2
Source: Cell Res. 2021 Jul 15;31(9):1011–23. doi: 10.1038/s41422-021-00531-8 (PMC8280646; doi:10.1038/s41422-021-00531-8)

Supplementary information, Fig. S4. Potent antigen presentation, Tfh, and GC generation. (a and b) C57BL/6 mice (n=7 or 8/group) were intramuscularly injected 1 nmole of I-E-F (mouse IFNα-eGFP-Fc) or eGFP. Four hours after injection, mice were sacrificed. Lymphocytes from mouse iLNs were collected to analyze the capture of I-E-F or eGFP. Flow cytometry analysis was proceeded to determine the percentages of GFP-positive macrophages ($B220^{-}CD11b^{+}F4/80^{+}$). The results of GFP-positive macrophages **(a)** and the representative flow cytometric contour plots **(b)** were present. The representative flow cytometric histograms related to Fig. 4e is shown in **(c)**. **(d-f)** Representative gating strategy for evaluating RBD-specific GC B cells, Tfh cells, and $OX40^{+}CD25^{+}$ Tfh cells related to Fig. 4f-j.

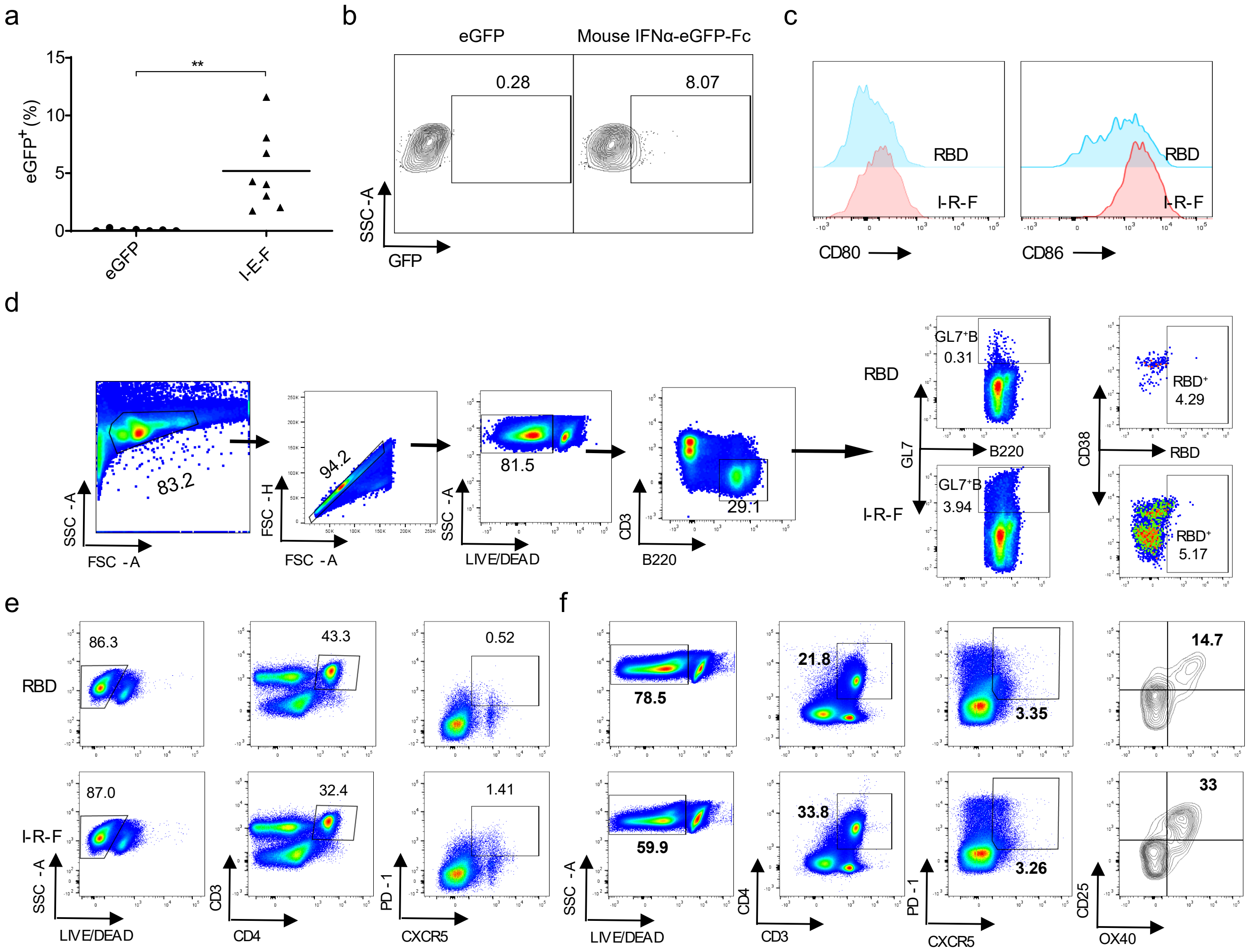

Supplement: Supplementary file 4 — Supplementary information, Fig. S4 [file 41422_2021_531_MOESM4_ESM.pdf]
