# Supplementary material for: Interferon-armed RBD dimer enhances the immunogenicity of RBD for sterilizing immunity against SARS-CoV-2
Source: Cell Res. 2021 Jul 15;31(9):1011–23. doi: 10.1038/s41422-021-00531-8 (PMC8280646; doi:10.1038/s41422-021-00531-8)

Supplementary information, Fig. S5. Design and characterization of the I-P-R-F (mouse), I-R-F (human), and I-P-R-F (human) vaccines. (a) The Schematic diagram of the I-P-R-F fusion protein. The showing structural elements contain a mouse IFNα4a (IFNα), $(G4S)_4$ linker (linker), Pan DR epitope (Pan), receptor-binding domain (RBD), immunoglobulin Fc fragment (Fc). **(b)** Size exclusion chromatography of mouse I-P-R-F, human I-R-F, and human I-P-R-F was performed on a Superdex200 Increase Column. The ultraviolet absorption at 280 nm is shown. The insert pictures show the SDS-PAGE of the eluted samples. **(c)** Real-time association and dissociation profiles of RBD, RBD-Fc, mouse I-R-F, mouse I-P-R-F, human I-R-F, and human I-P-R-F bound to hACE2. The KD value was calculated by the software BIA evaluation Version 4.1 (GE Healthcare). **(d)** The bioactivity of IFNα contained in mouse I-P-R-F was measured by an anti-viral infection biological assay.

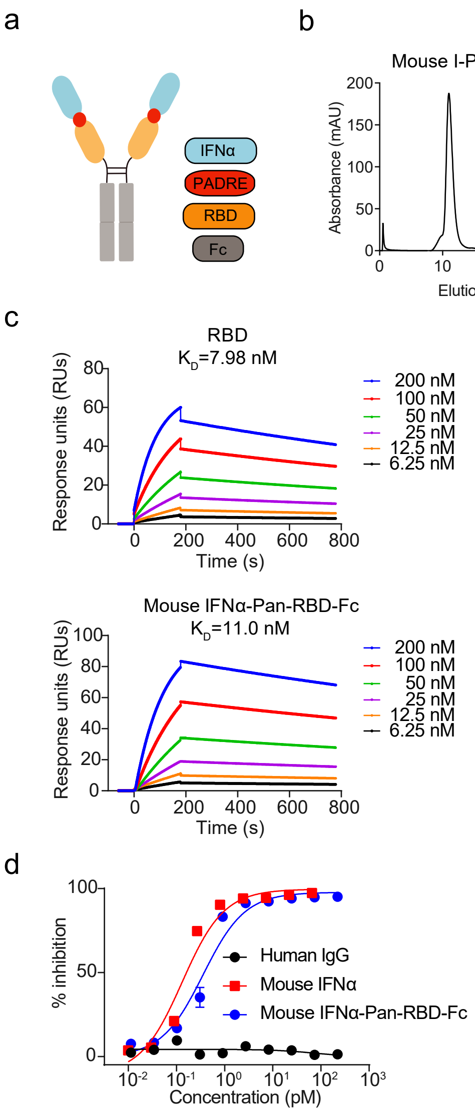

Supplement: Supplementary file 5 — Supplementary information, Fig. S5 [file 41422_2021_531_MOESM5_ESM.pdf]
