# Supplementary material for: Interferon-armed RBD dimer enhances the immunogenicity of RBD for sterilizing immunity against SARS-CoV-2
Source: Cell Res. 2021 Jul 15;31(9):1011–23. doi: 10.1038/s41422-021-00531-8 (PMC8280646; doi:10.1038/s41422-021-00531-8)

Fig. S6. The immunogenicity and protective efficacy of I-P-R-F in rhesus macaques. Male and female rhesus macaques (n=8) were equally divided into four groups with a sex ratio of 1:1 and were immunized i.m. with a high dose (50 μg) or a low dose (10 μg) of I-P-R-F with or without alum as an adjuvant and boost with the same dose at a 14-day interval. The sera were collected every week and analysed for I-P-R-F immunogenicity and vaccine-induced neutralizing antibodies. **(a)** RBD-specific IgG titers in the high-dose group were determined by ELISA at indicated time points. **(b)** The dynamic IgG titers in sera from the low-dose vaccinated group were determined. **(c-d)** The kinetics of neutralization antibody titers in serum from I-P-R-F immunized animals were determined by SARS-CoV-2 pseudovirus and authentic virus neutralization assays. **(e)** Neutralization of SARA-CoV-2 pseudovirus by the anti-sera from I-P-R-F immunized rhesus macaques before the virus challenge. **(f)** Viral load in anal swabs of rhesus macaques challenged with live SARS-CoV-2. **(g)** Viral load in the lungs. For each group of vaccinated macaques, 84 specimens from fourteen lung lobes were collected on day 7 post infection and subjected to determine the viral loads in the lungs. High dose/A: immunization with high dose with adjuvant, High dose/A-: immunization with high dose without adjuvant, Low dose/A: immunization with low does with adjuvant, Low dose/A-: immunization with low does without adjuvant. The dashed line indicates the limit of detection. Data are shown as mean ± SEM. *P*-values in (d) were analyzed with the unpaired t-test. *P*-values were calculated by one-way ANOVA with multiple comparisons tests in (e). ns (not significant), *$P < 0.05$, **$P < 0.01$, ***$P < 0.001$, ****$P < 0.0001$.

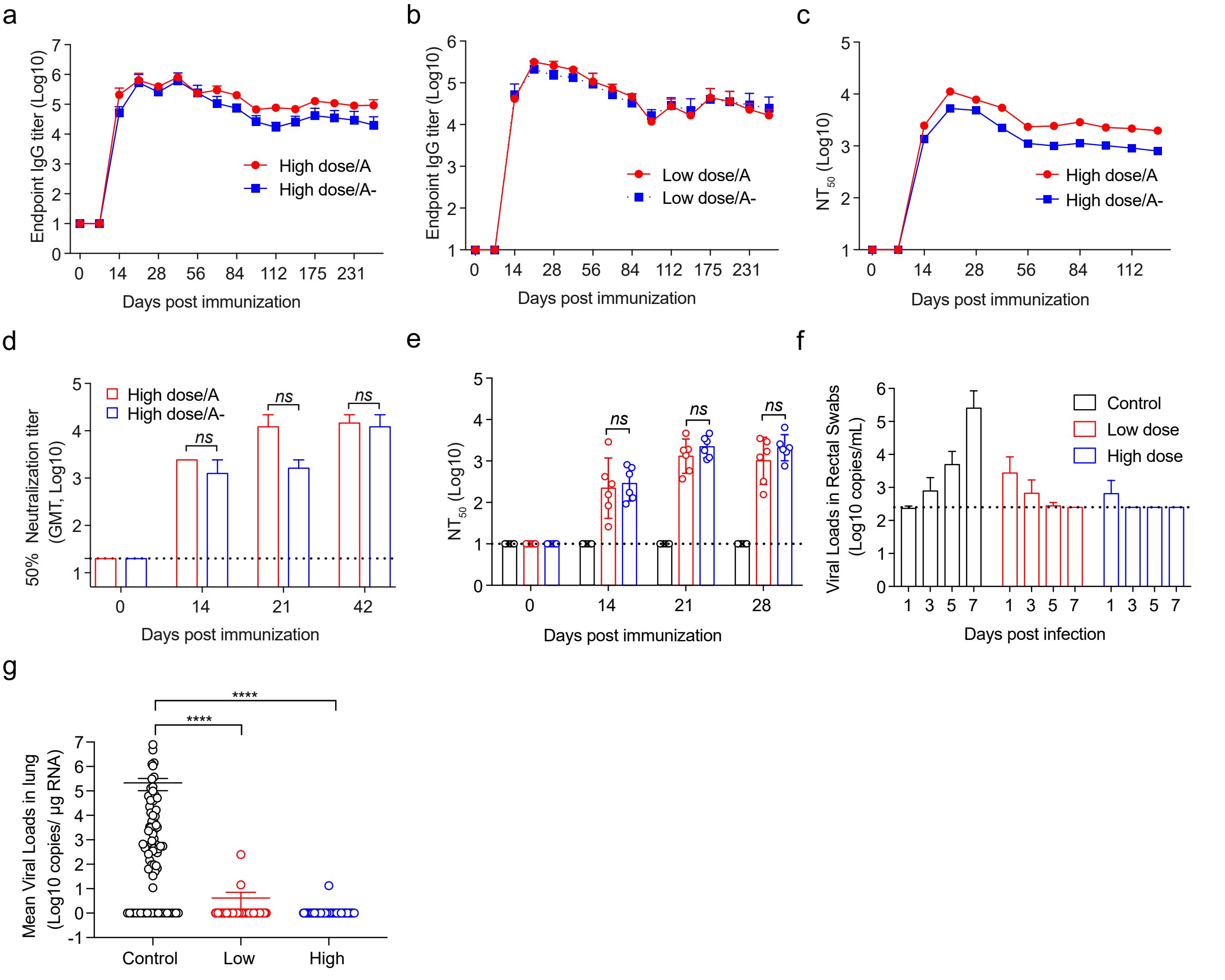

Supplement: Supplementary file 6 — Supplementary information, Fig. S6 [file 41422_2021_531_MOESM6_ESM.pdf]
